# Genomic Analysis of *Aeromonas salmonicida* ssp. *salmonicida* Isolates Collected During Multiple Clinical Outbreaks Supports Association with a Single Epidemiological Unit

**DOI:** 10.3390/pathogens13100908

**Published:** 2024-10-17

**Authors:** Konrad Wojnarowski, Paulina Cholewińska, Peter Steinbauer, Tobias Lautwein, Wanvisa Hussein, Lisa-Marie Streb, Dušan Palić

**Affiliations:** 1 Chair for Fish Diseases and Fisheries Biology, Faculty of Veterinary Medicine, Ludwig-Maximilians-Universität München, 80539 München, Germany; k.wojnarowski@lmu.de (K.W.); p.cholewinska@lmu.de (P.C.); wanvisa.hussein@gmail.com (W.H.); 2 Tiergesundheitsdienst Bayern e.V., 85586 Poing, Germany; peter.steinbauer@tgd-bayern.de; 3 Genomics & Transcriptomics Laboratory, Biological and Medical Research Centre (BMFZ), Heinrich Heine University Düsseldorf, 40225 Düsseldorf, Germany; tobias.lautwein@hhu.de; 4 Helmholtz Munich, Research Unit Comparative Microbiome Analysis, 85764 Neuherberg, Germany; lisamarie.streb@helmholtz-munich.de

**Keywords:** infection, disease, salmonids, third-generation sequencing, phylogeny analysis, furunculosis, Oxford Nanopore Technologies, autogenous vaccines, epidemiological unit, epidemiological link

## Abstract

Outbreaks of furunculosis cause significant losses in salmonid aquaculture worldwide. With a recent rise in antimicrobial resistance, regulatory measures to minimize the use of antibiotics in animal husbandry, including aquaculture, have increased scrutiny and availability of veterinary medical products to control this disease in production facilities. In such a regulatory environment, the utility of autogenous vaccines to assist with disease prevention and control as a veterinary-guided prophylactic measure is of high interest to the producers and veterinary services alike. However, evolving concepts of epidemiological units and epidemiological links need to be considered during approval and acceptance procedures for the application of autogenous vaccines in multiple aquaculture facilities. Here, we present the results of solid-state nanopore sequencing (Oxford Nanopore Technologies, ONT) performed on 54 isolates of *Aeromonas salmonicida* ssp. *salmonicida* sampled during clinical outbreaks of furunculosis in different aquaculture facilities from Bavaria, Germany, from 2017 to 2020. All of the performed analyses (phylogeny, single nucleotide polymorphism and 3D protein modeling for major immunogenic proteins) support a high probability that all studied isolates belong to the same epidemiological unit. Simultaneously, we describe a cost/effective method of whole genome analysis with the usage of ONT as a viable strategy to study outbreaks of other pathogens in the field of aquatic veterinary medicine for the purpose of developing the best autogenous vaccine candidates applicable to multiple aquaculture establishments.

## 1. Introduction

While salmonid aquaculture accounts for 3.5% [1] in weight, its economic value is at least valued at 10% of the global aquaculture production. Salmonid farming in Europe constitutes up to 30% of continental aquaculture production, highlighting its importance both as a food source and as a strong driver of a sustainable blue economy and value for society in general.

Diseases caused by pathogens are one of the most important factors contributing to losses in global aquaculture. Industry globalization allowed different pathogenic bacteria to spread beyond the regions of their initial occurrence. One of the fish pathogen clusters of high importance for both European and global aquaculture belongs to the genus *Aeromonas*, consisting of 17 to over 30+ species (the exact number still under debate, depending on the source) [2,3,4,5]. With this level of uncertainty, even for the actual number of the species present in the genus, there is an even bigger problem in establishing standardized or universal division criteria for different strains or isolates in the case of the same bacterial species belonging to the Aeromonads. The issue of differentiation of *Aeromonas sp.* strains and isolates becomes of great importance, especially in applying prophylactic measures based on autogenous vaccines in fish farming [6].

One of the most significant pathogens for salmonid aquaculture is *Aeromonas salmonicida* subspecies *salmonicida* (*A. s. s. s*). This bacterium belongs to the Gram-negative group from Proteobacteria phylum, which was discovered for the first time in a Bavarian brown trout hatchery by Emmerich and Weibel in 1894, and it was apparently present in the European salmonid aquaculture much prior to that date [7]. Besides salmonids, *Aeromonas salmonicida* possesses the ability to infect a wide array of hosts, which includes cyprinids, perches, pikes, flounders and some catfish. *Aeromonas salmonicida* is the causative agent for a disease called furunculosis. Furunculosis symptoms may vary depending on a number of different factors, i.e., species of host, environmental conditions, health state of the host, etc. [8].

Due to the continued presence of this bacteria in the environment and the continuing attempts to eradicate this pathogen with conventional approaches, including antibiotic treatments, the risk of growing antimicrobial resistance has increased, and eradication does not seem to be achievable. Therefore, strong biosecurity programs utilizing preventive measures such as vaccinations to boost fish immune systems to prevent infections are economically achievable and show promise [7]. Recent studies suggest that the widely recognized problem of growing antimicrobial resistance genes may be not only due to the effect of antibiotics [9] but also due to other drugs with antimicrobial properties [10]. This situation unquestionably indicates the importance of prevention rather than focusing solely on treatment strategies. One way of prevention was proposed by Sir Almroth Edward Wright back in 1903—autogenous vaccine, a concept which has not attracted much attention outside of the veterinary medicine area and for a long time was regarded as showing varying and inconsistent results [11,12].

Recently, due to ongoing advancements, manifesting both in the relative ease of use and economical approachability, scientists working on the topics of medical and veterinary prevention have gained a new ally—third-generation sequencing. Implementation of this technique in the area of aquatic veterinary medicine and autogenous vaccine production seems to have opened a new landscape, which will allow not only for the reduction in costs but also improve the safety and efficacy of autogenous vaccines [13]. At the same time, the results coming from the implementation of this novel technology might potentially affect the current trend regarding the usage of autogenous vaccines in veterinary medicine. One of the leading new implementations of third-generation sequencing is solid-state nanopore technology, which is currently represented by the Oxford Nanopore Technology (ONT). With recent improvements, ONT achieved a modal raw read accuracy of >99% using the new Q20+ chemistry and improved base-calling software, which brought the ONT in the range of Illumina sequencing that was regarded as a “golden standard” in medical applications [14]. Considering recent advances in ONT usage in clinical studies [15,16], nanopore sequencing can be regarded as a viable technique for usage in veterinary medicine, specifically autogenous vaccine production.

In this study, bacteria isolated during clinical furunculosis outbreaks in several salmonids: Alsatian char (*Salvelinus alpinus* × *fontinalis*), Brook trout (*Salvelinus fontinalis*), Arctic char *(Salvelinus alpinus*), Brown trout (*Salmo trutta*), Rainbow trout (*Oncorhynchus mykiss*) and Danube salmon (*Hucho hucho*), were sub-cultured after confirming them as *Aeromonas salmonicida* ssp. *salmonicida*. Isolates were subjected to ONT 3rd generation sequencing, and phylogenetic and structural comparison of major immunogenic proteins [17,18,19,20,21,22,23,24,25,26,27,28] analyses were performed. Additionally, the presence of single nucleotide polymorphisms (SNPs) was compared between the studied sequences to determine their similarity as further evidence to support the origin of the isolates from a single epidemiological unit in the geographical area of Bavaria. Our secondary goal was to create a cost-efficient and rapid pathogen identification and whole genome analysis method applicable to field studies in an effort to assist aquatic veterinarians and other fish health professionals to better understand the disease epidemiology and support evidence-based use of counter-measures against dynamic phenomena such as pathogen outbreaks in aquaculture.

## 2. Materials and Methods

### 2.1. Sample Collection

**Figure 1:**
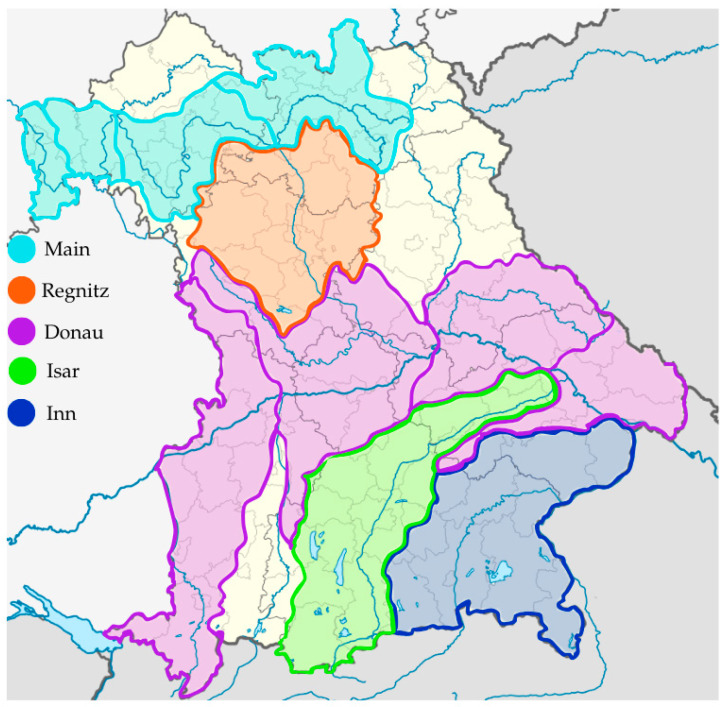
Map presenting watersheds where samplings were conducted in the timespan of 2017–2020.

The investigated *A. salmonicida* subspecies *salmonicida* isolates were collected from symptomatic Alsatian char, Brook trout (*Salvelinus alpinus* × *fontinalis*), Arctic char, Brown trout, Rainbow trout and Danube salmon specimens during disease outbreaks presenting with clinical symptoms typical for furunculosis. The samples were collected from several aquaculture establishments in the region of Bavaria, Germany, between 2017 and 2020. Sampling locations were located within watersheds of the Danube, Regen, Inn, Isar and Main as adjacent rivers (Figure 1). The fish samples were delivered to the Bavarian Animal Health Service, Fish Health Service division (Tiergesundheitsdienst Bayern e.V./Fischgesundheitsdienst, Poing, Germany) as a part of a routine laboratory diagnosis/investigation of clinical cases reported by the fish farmers. The initial analysis of the taxonomical status of collected isolates, proving their status as *Aeromonas salmonicida* subsp. *salmonicida*, was performed by Bavarian Animal Health Service, Fish Health Service division, with MALDI-TOF analysis.

### 2.2. Culture of Aeromonas salmonicida subsp. salmonicida

Bacterial isolates were stored in the Cryotube system and reactivated using Colombia blood agar plates; then, additional traits such as shape (round) and positive beta-hemolytic activity of the cultured bacteria were checked, and positive colonies were used in subsequent sub-cultures. Verified isolates were liquid cultured for 3–4 days using Double-Strength Tryptic Soy Broth (Merck, Darmstadt, Germany) to achieve a sufficient number of microorganisms necessary for isolating DNA material of both plasmid and genomic origin. All cultures were performed at 20 °C, as per the findings of Stuber et al. [29]. For each of the isolates, a liquid culture was set up in duplicate in order to extract genomic and plasmid DNA.

### 2.3. DNA Extraction

Plasmid and genomic DNA extraction was performed with Nucleospin Microbial DNA and Nucleospin Plasmid kits (Macherey-Nagel, Dueren, Germany) according to the manufacturer’s protocols. After extraction, genomic DNA samples were additionally cleaned with Nucleospin gDNA Clean-kit (Macherey-Nagel, Germany) according to the manufacturer’s protocol. Prior to the library preparation, samples were pooled in a 1:1 ratio. The resulting concentrations of genomic and plasmid DNA were then checked using a Qubit™ 4 Fluorometer (Thermo Fisher Scientific, Waltham, MA, USA) and were in the range of 55–60 ng/µL. In turn, the quality of the samples was checked using a Nanodrop 1000 Spectrophotometer (Thermo Fisher Scientific, USA); the absorbance parameters for the samples tested were 260/230:2-2.2 and 260/280:1.8.

### 2.4. Library Preparation and Sequences Analysis

Whole genome sequencing (WGS) was performed using the Oxford Nanopore system (Oxford PromethION) by Genomics & Transcriptomics Labor Düsseldorf (Heinrich-Heine-Universität Düsseldorf, Düsseldorf, Germany). Sequences were analyzed on the Galaxy web platform [30]). FastQC and NanoPlot were used to check the quality. Next, the Flye command was used, followed by a results check with Fasta statistics. Checked sequences were mapped to the reference genome by Map with minimap2 (reference genome: *Aeromonas salmonicida* subsp. *salmonicida* A449—GenBank CP000644.1). The data were polished using the Racon command and summarized by Quast. The next step was to compare all samples with each other to check for the existence of differences between each bacterial isolate using BLAST (NCBI BLAST+ blastn). For SNP analysis, Clair3, bcftools consensus and SnpSift Filter were used as suggested by Batut et al. [31]

Additionally, to check the presence of virulence and antimicrobial genes in samples, the ABRiacte command with VFDB [32] and CARD [33] databases were utilized.

To prepare the phylogenetic tree, Clair3, ClustalW, bfctools and Newick Display at Galaxy web page (https://usegalaxy.org, accessed on 20 January 2024)) were used, and for visualization of the data, the microreact web page (https://microreact.org, accessed on 20 January 2024)) was used [34].

Protein structure analysis was performed using blastp (sequences were translated using blastx) and the neurosnap web page (https://neurosnap.ai, accessed on 20 January 2024) with 3D modeling performed by usage of Alphafold2 [35].

## 3. Results

The average level of bp in samples was 4,989,773 (+/− 145,582) and G+C content of 57.65% (+/− 4.03). In the case of similarity to the *Aeromonas salmonicida* subspecies *salmonicida* A449 strain, the mean result was 97.83% (+/− 0.16) (Table 1). The tested samples from the Bavaria region in Germany did not show any significant differences in the identity level in comparison to the reference genome or in the case of G + C content. Additionally, no correlation was found between the differences in genome sequences, G+C content, and time or place of the sampling.

Virulence genes that were present in all studied samples are presented in Table 2, namely the following: VPA0450 [type III secretion system effector; T3SS1 (VF0408); accession number: NP_799960] and pscR [type III secretion system protein PscR; TTSS (VF0083); accession number: NP_250384]. Regarding the parameters of the analysis, the detected coverage was higher than 99%, and the identity between the samples was higher than 82%.

Additionally, analysis using the CARD database evidenced the presence of genes such as MCR-3 (resistance to peptide), FOX-2 (resistance to cephalosporin, cephamycin), cphA5 (resistance to carbapenem) and OXA-427 (resistance to cephalosporin, penam) were found. MCR-3 is a plasmid-borne phosphoethanolamine transferase and has been described by Yin et al. [36] from a porcine *Escherichia coli* plasmid pWJ1. Similar to it is FOX-2, which is a beta-lactamase that has been found for the first time in *E. coli* (Table 2).

**Figure 2:**
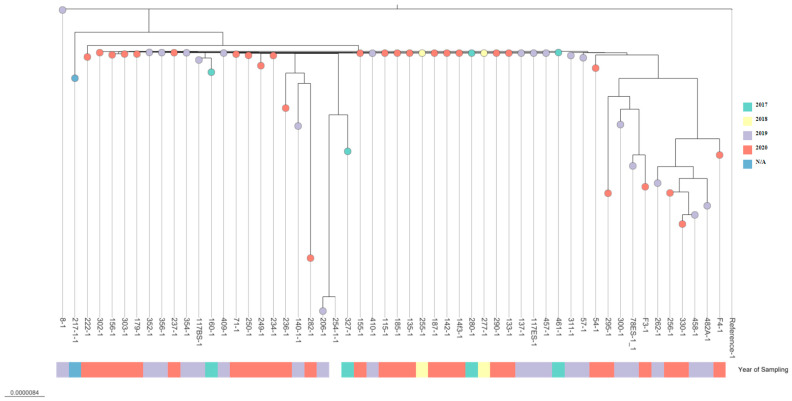
Phylogenetic tree of *Aeromonas salmonicida* ssp. *salmonicida* isolates. The scale bar indicates the evolutionary distance in substitutions per nucleotide. The tree was visualized using microreact.org.

Phylogenetic analysis clearly demonstrated a lack of significant differences between the time of sampling or geographical location in regard to the isolate’s genetic makeup. The highest genetic distance has been observed between samples: 54-1, 206-1, 217-1, 236-1, 254-1, 256-1, 262-1, 295-1, 300-1, 327-1, 330-1, 482A-1, 78ES-1_1, F3-1 and F4-1. The genetic distance in this case was about 5.1e^−8^ to 9.4e^−6^ in comparison to the reference sequence. In the case of other samples, genetic distance was from 2.1e^−7^ to 4.9e^−8^, where the least distance occurred in the case of samples 8-1 and 217-1 (less than 2.1e^−8^) (Figure 2).

**Figure 3:**
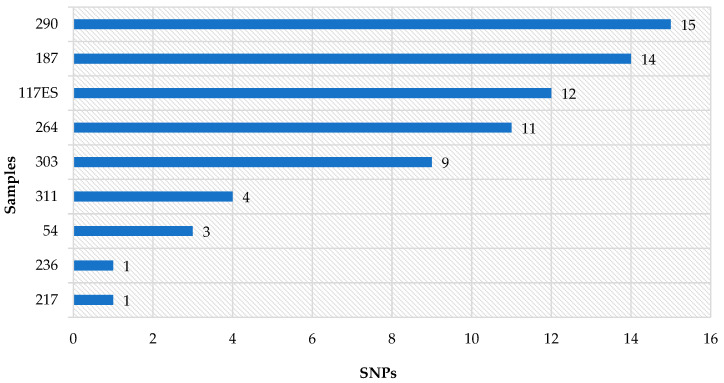
Results of the SNP analysis representing differences in the number of single nucleotide polymorphisms in tested samples (other samples = 0 SNP difference). (Analysis was performed using the Galaxy web platform and SNP distance matrix 0.8.2 tool).

In the case of SNPs, tested samples were characterized by values from 0 to 15. The highest level was detected in samples 290-1; 187-1, 254-1 and 117ES-1 (10–15 SNPs); the rest of the samples had four or fewer SNPs (Figure 3).

**Figure 4:**
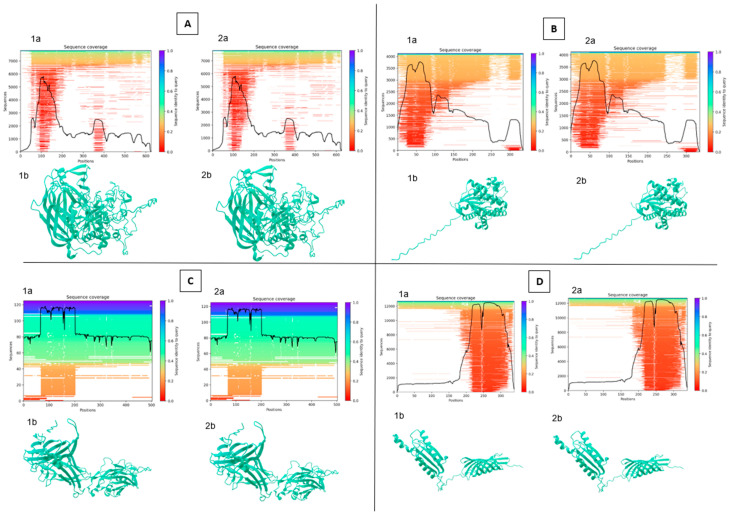

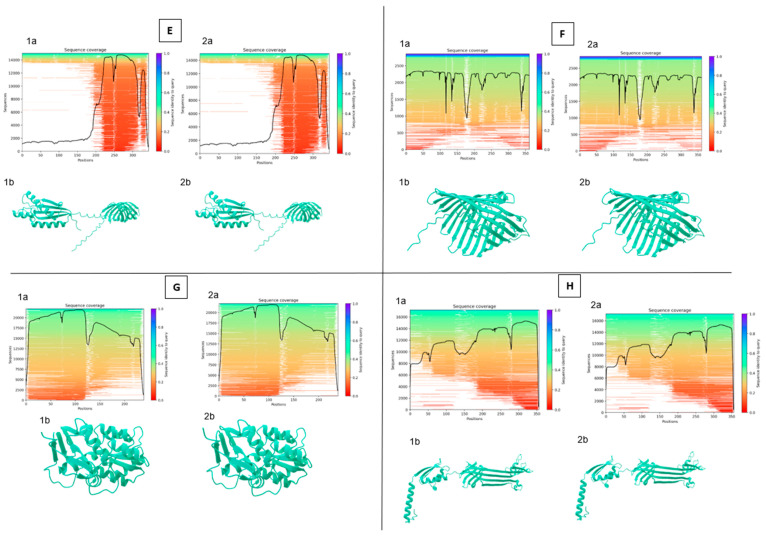

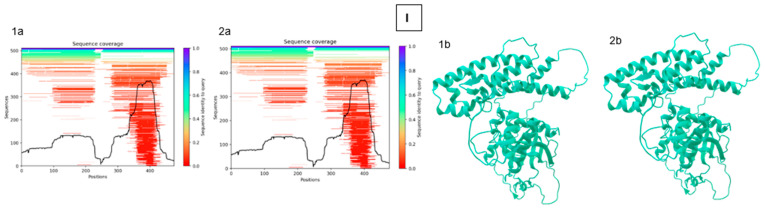
Protein structure analysis between reference and selected sample (290-1) [(**A**)—ASA_2540; (**B**)—ASA_ 0509; (**C**)—ASA_1438; (**D**)—ASA_1267; (**E**)—OmpA; (**F**)—OmpC; (**G**)—OmpF; (**H**)—AscC; (**I**)—AexT; a—sequencing coverage; b—3D structure; 1—sample; 2—reference] (analysis was performed using Neurosnap platform and Alphafold 2 model).

In the case of 54 sequences that have met all quality conditions, the nucleotide, amino acid and 3D structures were compared with each other and with A449 *Aeromonas salmonicida* subsp. *salmonicida* reference strain. Nine proteins with high immunogenic potential, as determined by the review of the scientific literature, were selected for further studies [17,18,19,20,21,22,23,28,37,38,39]. No differences in amino acid or protein structure were found between all studied isolates sampled across Bavaria. Three-dimensional modeling performed by the usage of Alphafold2 has proposed that the structure of all the listed proteins is most likely identical, thus confirming the possibility that the same mechanisms and immunological responses will be involved during the infection processes. Due to all isolates having the same protein composition, 3D modeling was only performed for the A290 isolate (Figure 4 and Figure S1, Table S1).

## 4. Discussion

Analyses of genomic structures suggest a high degree of probability that all tested *A. s. s. s.* isolates belong to the same epidemiological unit within Bavaria, Germany. The presence of minor/insignificant genomic differences within the tested pool of *A. s. s. s.* isolates is consistent with the ongoing debate in the scientific community regarding the importance and meaning of genetic differences in the taxonomy of this bacterium [40,41,42,43].

In the available literature, there is a high genetic similarity between different *A. salmonicida* strains, which has been evidenced by Dallaire-Dufresne [44], where the 01-B526 strain was found to be highly similar to the previously described A449, with the difference being found only in the plasmid content. Is it a widely accepted fact that most of the differences detected in the case of the *A. salmonicida* strains can be attributed to the genetic material coming from the plasmidome. In a review by Vincent et al. [45], it is stated that one of the key factors in changes taking place in the genetic material of *A. salmonicida* is Horizontal Gene Transfer and that such information can be used to determine the ecology of the isolates. *A. salmonicida* strains frequently display antibiotic resistance, usually due to the presence of plasmids that carry antibiotic and heavy metal resistance genes, supporting our findings of the presence of the same AMR genes (namely MRC-3, FOX-2, cphA5 and OXA-427) in all of the studied isolates.

Analysis of the available *Aeromonas* sp. genomes performed by Studer et al. [46] demonstrated their relative uniformity. About 70% of the open reading frames (ORFs) are shared by all *Aeromonas* spp., and about 20% are shared by at least two species, meaning that only 10% of the ORFs are specific to a given species. Some of the studies have even suggested that, because of the DNA–DNA hybridization on a level of close to 76% between *Aeromonas* family representatives, namely *A. salmonicida* and *A. bestiarum*, they may, in reality, represent a single taxon [47].

Some of the recent studies performed by Pradhan et al. [48], in which authors were studying a wide range of characteristics related to the pathogenicity of *A. s. s. s.* bacterium isolated from three Indian native fish species (*Labeo rohita*, *Labeo catla* and *Cirrhinus mrigal*) have also evidenced significant uniformity among these isolates. The authors have constructed a phylogenetic tree based on a partial 16s rRNA gene sequence, and no differences between the four reference isolates and the studied isolate were discovered. Although the results obtained in the case of our studies cannot be directly compared to the data presented by Pradhan and his team, due to the differences in methodology and the source of the data, it is important to consider that 16s rRNA regions tend to be highly conserved, and that differences between strains are more likely to be detected in plasmid or genomic content, further suggesting that resolution of our studies allows for identity comparison with high certainty. Similarly, in studies by Long et al. [42], 25 isolates of *Aeromonas salmonicida* were subjected to ANI analysis (average nucleotide identity), and their strains were found to be similar over 96,5% upwards, whereas in the case of our samples, average similarity between the studied isolates was on the level of 99,933%, strongly suggesting that our collection of isolates are almost identical, and therefore likely originate from the same epidemiological unit.

Further evidence that *Aeromonas salmonicida* ssp. *salmonicida* isolates are highly similar to each other comes from the studies by Bartkova et al. [49]. In said studies, 99 Danish, 1 Scottish and 1 NCIMB 1102 strains were compared. Samples were compared based on the analysis of 667 SNPs, and in the result, the biggest detected difference was found to be 147 SNPs. In the case of their comparison to the A449 reference strain, which was also used as the reference in our study, the detected difference was on the level of <15 SNPs. Another important piece of information in Bartkova et al.’s [49] studies is the association of the number of SNPs detected between the analyzed strains and the year of sampling, which shows that time may not be the most influential factor driving genetic changes in *A. salmonicida*. It is also of interest to note that in the studies performed by Reith [27], the *A. salmonicida* reference strain A449 exhibited a number of changes from its closely related *A. hydrophila*, where the author stated that its 9% “difference in gene content indicating instances of single gene or operon loss or gain”. Those differences are most likely related to the adaption of *A. salmonicida* to the host salmonids, compared to *A. hydrophila*, which has a significantly wider range of potential hosts.

An additional factor indicating the similarity of the isolates studied in this publication, apart from the high similarity of the previously mentioned nucleotide and amino acid sequences, is the three-dimensional structure of proteins similarity predicted by Alphafold 2. This assumption is strengthened by evidence coming from the available literature. Studies by Gao et al. [50], where a DNA vaccine encoding the ompA gene (one of the genes with high immunogenic potential) was administered to the mice, resulted in strong immune response against *Aeromonas veronii*, confirming the importance of this gene for *Aeromonas veronii* virulence, as well as virulence in its close relatives, such as *Aeromonas salmonicida*. This fact highlights the meaning of our results regarding the shared OmpA gene, protein and structural makeup between analyzed Bavarian isolates. Another highly important gene that was a subject of our analysis was the AscC gene, which encodes the outer membrane pore of the secretion apparatus in a number of *Aeromonas* species. AscC gene, along with its effector AexT gene, plays a crucial role in the work of the type III secretion system (TTSS), which has been evidenced in a number of studies to be a major, if not the most important virulence and immunogenicity gene for *Aeromonas* spp. [51,52,53]. This key role of TTSS and genes involved with it in *Aeromonas salmonicida* are located on the pAsa5 plasmid, unstable at higher temperatures, confirmed by bacteria losing their TTSS and becoming avirulent when cultivated at higher temperatures [54,55]. Similar to the above, in our isolates, the gene and amino acid structure predicted by Alphafold 2 were identical, implicating that the immune response against the studied isolates would likely be very similar.

The results of the aforementioned studies corroborate with our results and indicate that major immunogenic proteins and genetic structure of *Aeromonas salmonicida* isolates are highly conservative, thus strengthening the possibility that autogenous vaccines created from consensus isolate (exhibiting the highest average degree of similarity to the other isolates in the present study) would be a viable strategy of cost- and time-effective prophylaxis against future outbreaks of furunculosis in farmed salmonids in the area of Bavaria, Germany. Furthermore, the results of our research and the analysis of the literature indicate that in the case of *Aeromonas salmonicida* subsp. *salmonicida*, we are dealing with a bacterium characterized by the relative stability of genetic material of genomic origin (last 50 years) [49], and at the same time, characterized by extraordinary plasticity in terms of the ability to accept plasmids depending on the conditions of various gene expression. Such threat becomes a concern due to the increased risk of emerging infections in naïve fish populations and due to the risk of bypassing mechanisms related to the action of biocides (antimicrobials). Therefore, it is highly recommended that further research be conducted aimed at producing autogenous vaccines based on thorough, rapid, and cheap genomic and bioinformatics analysis of the epidemiological situation using the latest technologies and knowledge to protect fish populations against this pathogen.

## 5. Conclusions

The results of the performed comprehensive analysis indicate that the *A. salmonicida* subsp. *salmonicida* isolates examined in this study originate from the same epidemiological unit. At the same time, the cost-effective and rapid WGS analysis method developed for this study, combined with data on the amino acid sequence of the main immunogenic proteins and the prediction of their 3D structure, can be used in the future for the analysis of other bacteria for the purposes of the evidence-based selection for the best available candidate for producing an autogenous vaccine for different aquaculture establishments within a single epidemiological unit, or with a confirmed epidemiological link [13].

## Figures and Tables

**Table 1:** Level of G+C content, % identity and numbers of bp for each tested sample (analysis performed using Galaxy web platform with geecee 5.0.0 tool).

Sample_ID	GC_ Content	% of Identity	No bp	Sample_ID	GC_ Content	% of Identity	No bp
280-1	58.07	97.82	5,102,882	249-1	58.14	97.86	5,170,112
133-1	58.31	97.98	4,193,552	237-1	58.15	97.86	5,097,470
8-1	58.37	97.78	4,877,049	236-1	58.29	97.92	4,946,207
F4-1	58.07	97.85	5,124,310	234-1	58.10	97.98	5,156,757
F3-1	58.07	97.87	5,123,926	222-1	58.32	97.01	4,705,879
482A-1	58.32	97.85	4,874,581	217-1	58.29	97.75	4,903,569
461-1	58.11	97.81	5,090,948	206-1	58.11	97.86	5,089,959
458-1	58.26	97.83	4,960,305	187-1	58.28	97.83	4,996,262
457-1	58.20	98.11	5,041,256	179-1	58.19	97.82	4,958,944
410-1	58.21	97.81	4,952,850	160-1	58.32	97.71	4,874,696
409 -1	58.17	97.82	5,010,907	156-1	58.20	97.81	5,018,443
356-1	58.26	97.88	5,002,169	155-1	58.29	97.81	4,923,057
354-1	28.33	97.90	4,958,302	142-1	58.26	97.84	4,944,355
352-1	58.19	97.83	5,059,069	140-1	58.29	97.85	4,979,227
330-1	58.30	97.84	4,908,635	137-1	58.19	98.00	5,016,263
327-1	58.13	97.78	5,092,954	135-1	58.39	97.85	4,874,196
311-1	58.31	98.00	4,957,054	117ES-1	58.22	97.78	5,007,215
303-1	58.13	97.97	5,106,372	117BS-1	58.22	97.76	5,004,904
300-1	58.32	97.91	4,986,132	115-1	58.17	97.97	5,067,920
295-1	58.37	97.70	4,876,990	78ES-1	58.31	97.90	4,899,038
290-1	58.06	97.83	5,161,521	71-1	58.16	97.89	5,081,602
282-1	58.36	97.90	4,952,918	57-1	58.12	97.78	4,961,082
277-1	58.14	97.81	5,057,870	54-1	58.15	97.82	5,039,876
262-1	58.15	97.76	5,057,594	14f3-1	58.16	97.86	5,024,076
256-1	58.09	97.81	5,101,717	302-1	58.07	97.83	5,102,887
255-1	58.38	97.91	4,877,049	185-1	58.15	97.85	5,022,918
250-1	58.09	97.82	5,194,870	254-1	58.00	97.22	4,877,053

**Table 2:** Virulence and antimicrobial genes found in analyzed *Aeromonas salmonicida* subsp. *salmonicida* (analysis performed on Galaxy web platform using Abricate 1.0.1 tool).

Genes	Resistance	Other Information	Accesion Number	References	Data Base
MCR-3	Peptide	MCR-3 is a plasmid-borne phosphoethanolamine transferase that interferes with binding of colistin to the cell membrane via addition of phosphoethanolamine to lipid A, resulting in a reduction in negative charge of the cell membrane. Originally described by Yin et al. 2017 from a porcine *Escherichia coli* plasmid pWJ1.	ARO:3004139	[36,37]	CARD
FOX-2	cephalosporin; cephamycin	FOX-2 is a beta-lactamase found in *Escherichia coli*	ARO:3002156	[38]	CARD
*cphA5*	Carbapenem	*cphA5* is an Ambler Class B MBL; subclass B2 originally isolated from *Aeromonas salmonicida*. This enzyme has specific activity against carbapenems and is active as a mono-zinc protein.	ARO:3003101	[39]	CARD
OXA-427	cephalosporin; penam	OXA-427 is a novel CHDL most closely related to chromosomal class D β-lactamase. It confers resistance to penicillins, ceftazidime, aztreonam and, in some instances, to carbapenems.	ARO:3007719	[40]	CARD
**Genes**	**Product**		**Accession Number**		**Data Base**
VPA0450	(VPA0450) type III secretion system effector [T3SS1 (VF0408)]		NP_799960		vfdb
*pscR*	(pscR) type III secretion system protein PscR [TTSS (VF0083)]		NP_250384		vfdb

## Data Availability

The data presented in this study are openly available in Figshare at https://figshare.com/s/6483bf06b38455bf7bd5?file=49507536 (accessed on 14 October 2024).

## Supplementary Materials

The following supporting information can be downloaded at: https://www.mdpi.com/article/10.3390/pathogens13100908/s1, Table S1: Comparison of reference protein with samples (blastp); Figure S1: Protein structure analysis between reference and selected sample (290-1) [A—ASA_2540; B—ASA_ 0509; C—ASA_1438; D—ASA_1267; E—OmpA; F—OmpC; G—OmpF; H—AscC; I—AexT; c—predicted LDDT per position; d—PAE; 1—sample; 2—reference].

## References

[1] FAO (2023). Top 10 Species Groups in Global Aquaculture 2021.

[2] Liu D. (2015). Aeromonas. Molecular Medical Microbiology.

[3] Sanglas A., Albarral V., Farfán M., Lorén J.G., Fusté M.C. (2017). Evolutionary roots and diversification of the genus Aeromonas. Front. Microbiol..

[4] Fernández-Bravo A., Figueras M.J. (2020). An update on the genus Aeromonas: Taxonomy, epidemiology, and pathogenicity. Microorganisms.

[5] Bertran X., Rubio M., Góme L., Llovet T., Muñoz C., Navarro F., Miro E. (2021). Taxonomic identification of different species of the genus Aeromonas by whole-genome sequencing and use of their species-specific β-lactamases as phylogenetic markers. Antibiotics.

[6] Barnes A.C., Rudenko O., Landos M., Dong H.T., Lusiastuti A., Phuoc L.H., Delamare-Deboutteville J. (2022). Autogenous vaccination in aquaculture: A locally enabled solution towards reduction of the global antimicrobial resistance problem. Rev. Aquac..

[7] Bernoth E.M., Ellis A.E., Midtlyng P.J., Olivier G., Smith P. (1997). Furunculosis: Multidisciplinary Fish Disease Research.

[8] Chong R.S.M. (2022). Furunculosis. Aquaculture Pathophysiology.

[9] Guillen N., Li C., Rosener B., Mitchell A. (2024). Antibacterial activity of nonantibiotics is orthogonal to standard antibiotics. Science.

[10] Elbaz M., Stein E., Raykhshtat E., Weiss-Meilik A., Cohen R., Ben-Ami R. (2023). Exposure to Non-Antimicrobial Drugs and Risk of Infection with Antibiotic-Resistant Enterobacteriaceae. Antibiotics.

[11] Walker I.C. (1917). Study III: Studies on the Sensitization of Patients with Bronchial Asthma to Bacterial Proteins as Demonstrated by the Skin Reaction and the Methods Employed in the Preparation of These Proteins. J. Med. Res..

[12] Giedrys-Kalemba S., Czernomysy-Furowicz D., Fijałkowski K., Jursa-Kulesza J. (2018). Autovaccines in individual therapy of staphylococcal infections. Pet-To-Man Travelling Staphylococci.

[13] Behr K.P., Zeller G., Selbitz H.-J. (2023). Manual of Autogenous Vaccines (AV).

[14] Stoler N., Nekrutenko A. (2021). Sequencing error profiles of Illumina sequencing instruments. NAR Genom. Bioinform..

[15] Sheka D., Alabi N., Gordon P.M. (2021). Oxford nanopore sequencing in clinical microbiology and infection diagnostics. Brief. Bioinform..

[16] Mostafa H.H. (2024). An evolution of Nanopore next-generation sequencing technology: Implications for medical microbiology and public health. J. Clin. Microbiol..

[17] Vanden Bergh P., Heller M., Braga-Lagache S., Frey J. (2013). The *Aeromonas salmonicida* subsp. salmonicida exoproteome: Determination of the complete repertoire of Type-Three Secretion System effectors and identification of other virulence factors. Proteome Sci..

[18] Vanden Bergh P., Heller M., Braga-Lagache S., Frey J. (2013). The *Aeromonas salmonicida* subsp. salmonicida exoproteome: Global analysis, moonlighting proteins and putative antigens for vaccination against furunculosis. Proteome Sci..

[19] Vasquez I., Cao T., Hossain A., Valderrama K., Gnanagobal H., Dang M., Santander J. (2020). *Aeromonas salmonicida* infection kinetics and protective immune response to vaccination in sablefish (*Anoplopoma fimbria*). Fish Shellfish Immunol..

[20] Hirst I.D., Ellis A.E. (1994). Iron-regulated outer membrane proteins of *Aeromonas salmonicida* are important protective antigens in Atlantic salmon against furunculosis. Fish Shellfish Immunol..

[21] Yadav S.K., Meena J.K., Sharma M., Dixit A. (2016). Recombinant outer membrane protein C of *Aeromonas hydrophila* elicits mixed immune response and generates agglutinating antibodies. Immunol. Res..

[22] Yadav S.K., Sahoo P.K., Dixit A. (2014). Characterization of immune response elicited by the recombinant outer membrane protein OmpF of *Aeromonas hydrophila*, a potential vaccine candidate in murine model. Mol. Biol. Rep..

[23] Salte R., Norberg K., Arnesen J.A., Ødegaard O.R., Eggset G. (1992). Serine protease and glycerophospholipid: Cholesterol acyltransferase of *Aeromonas salmonicida* work in concert in thrombus formation; in vitro the process is counteracted by plasma antithrombin and α2-macroglobulin. J. Fish Dis..

[24] Chu S., Cavaignac S., Feutrier J., Phipps B.M., Kostrzynska M., Kay W.W., Trust T.J. (1991). Structure of the tetragonal surface virulence array protein and gene of *Aeromonas salmonicida*. J. Biol. Chem..

[25] Lutwyche P., Exner M.M., Hancock R.E., Trust T.J. (1995). A conserved *Aeromonas salmonicida* porin provides protective immunity to rainbow trout. Infect. Immun..

[26] Ebanks R.O., Goguen M., McKinnon S., Pinto D.M., Ross N.W. (2005). Identification of the major outer membrane proteins of *Aeromonas salmonicida*. Dis. Aquat. Org..

[27] Reith M.E., Singh R.K., Curtis B., Boyd J.M., Bouevitch A., Kimball J., Brown L.L. (2008). The genome of *Aeromonas salmonicida* subsp. salmonicida A449: Insights into the evolution of a fish pathogen. BMC Genom..

[28] Diao J., Li L., Fan Y., Wang S., Gai C., Wang Y., Ye H. (2020). Recombinant outer membrane protein C of *Aeromonas salmonicida* subsp. masoucida, a potential vaccine candidate for rainbow trout (*Oncorhynchus mykiss*). Microb. Pathog..

[29] Stuber K., Burr S.E., Braun M., Wahli T., Frey J. (2003). Type III secretion genes in *Aeromonas salmonicida* subsp. salmonicida are located on a large thermolabile virulence plasmid. J. Clin. Microbiol..

[30] Afgan E., Baker D., Van den Beek M., Blankenberg D., Bouvier D., Čech M., Goecks J. (2016). The Galaxy platform for accessible, reproducible and collaborative biomedical analyses: 2016 update. Nucleic Acids Res..

[31] Batut B., Nasr E., Zierep P. Pathogen Detection from (Direct Nanopore) Sequencing Data Using Galaxy—Foodborne Edition (Galaxy Training Materials). https://training.galaxyproject.org/training-material/topics/microbiome/tutorials/pathogen-detection-from-nanopore-foodborne-data/tutorial.html.

[32] Chen L., Zheng D., Liu B., Yang J., Jin Q. (2016). VFDB 2016: Hierarchical and refined dataset for big data analysis—10 years on. Nucleic Acids Res..

[33] Alcock B.P., Huynh W., Chalil R., Smith K.W., Raphenya A.R., Wlodarski M.A., Edalatmand A., Petkau A., Syed S.A., Tsang K.K. (2023). CARD 2023: Expanded curation, support for machine learning, and resistome prediction at the Comprehensive Antibiotic Resistance Database. Nucleic Acids Res..

[34] Argimón S., Abudahab K., Goater R., Fedosejev A., Bhai J., Glasner C., Feil E., Holden M., Yeats C., Grundmann H. (2016). Microreact: Visualizing and sharing data for genomic epidemiology and phylogeography. Microb. Genom..

[35] Jumper J., Evans R., Pritzel A., Green T., Figurnov M., Ronneberger O., Tunyasuvunakool K., Bates R., Žídek A., Potapenko A. (2021). Highly accurate protein structure prediction with AlphaFold. Nature.

[36] Yin W., Li H., Shen Y., Liu Z., Wang S., Shen Z., Zhang R., Walsh T.R., Shen J., Wang Y. (2017). Novel plasmid-mediated colistin resistance gene mcr-3 in Escherichia coli. MBio.

[37] Gonzalez-Avila L.U., Loyola-Cruz M.A., Hernández-Cortez C., Bello-López J.M., Castro-Escarpulli G. (2021). Colistin resistance in *Aeromonas* spp.. Int. J. Mol. Sci..

[38] Piotrowska M., Przygodzińska D., Matyjewicz K., Popowska M. (2017). Occurrence and variety of β-lactamase genes among Aeromonas spp. isolated from urban wastewater treatment plant. Front. Microbiol..

[39] Bottoni C., Marcoccia F., Compagnoni C., Colapietro M., Sabatini A., Celenza G., Segatore B., Maturo M.G., Amicosante G., Perilli M. (2015). Identification of new natural CphA metallo-β-Lactamases CphA4 and CphA5 in *Aeromonas veronii* and *Aeromonas hydrophila* isolates from municipal sewage in central Italy. Antimicrob. Agents Chemother..

[40] Bogaerts P., Naas T., Saegeman V., Bonnin R.A., Schuermans A., Evrard S., Bouchahrouf W., Jove T., Tande D., De Bolle X. (2017). OXA-427, a new plasmid-borne carbapenem-hydrolysing class D β-lactamase in Enterobacteriaceae. J. Antimicrob. Chemother..

[41] Janda J.M., Abbott S.L. (2010). The genus Aeromonas: Taxonomy, pathogenicity, and infection. Clin. Microbiol. Rev..

[42] Long M., Fan H., Gan Z., Jiang Z., Tang S., Xia H., Lu Y. (2023). Comparative genomic analysis provides insights into taxonomy and temperature adaption of *Aeromonas salmonicida*. J. Fish Dis..

[43] Charette S.J. (2023). *Aeromonas salmonicida*: Genomics, Taxonomy, Diversity, Pathogenesis, Treatments and Beyond. Microorganisms.

[44] Dallaire-Dufresne S., Tanaka K.H., Trudel M.V., Lafaille A., Charette S.J. (2014). Virulence, genomic features, and plasticity of *Aeromonas salmonicida* subsp. salmonicida, the causative agent of fish furunculosis. Vet. Microbiol..

[45] Vincent A.T., Hosseini N., Charette S.J. (2021). The *Aeromonas salmonicida* plasmidome: A model of modular evolution and genetic diversity. Ann. N. Y. Acad. Sci..

[46] Studer N., Frey J., Vanden Bergh P. (2013). Clustering subspecies of *Aeromonas salmonicida* using IS630typing. BMC Microbiol..

[47] Martínez-Murcia A.J., Soler L., Saavedra M.J., Chacón M.R., Guarro J., Stackebrandt E., Figueras M.J. (2005). Phenotypic, genotypic, and phylogenetic discrepancies to differentiate *Aeromonas salmonicida* from *Aeromonas bestiarum*. Int. Microbiol..

[48] Pradhan S.K., Devi R., Khan M.I.R., Kamilya D., Choudhury T.G., Parhi J. (2023). Isolation of *Aeromonas salmonicida* subspecies salmonicida from aquaculture environment in India: Polyphasic identification, virulence characterization, and antibiotic susceptibility. Microb. Pathog..

[49] Bartkova S., Leekitcharoenphon P., Aarestrup F.M., Dalsgaard I. (2017). Epidemiology of Danish *Aeromonas salmonicida* subsp. salmonicida in fish farms using whole genome sequencing. Front. Microbiol..

[50] Gao S., Zhao N., Amer S., Qian M., Lv M., Zhao Y., Zhao B. (2013). Protective efficacy of PLGA microspheres loaded with divalent DNA vaccine encoding the ompA gene of *Aeromonas veronii* and the hly gene of *Aeromonas hydrophila* in mice. Vaccine.

[51] Dacanay A., Knickle L., Solanky K.S., Boyd J.M., Walter J.A., Brown L.L., Reith M. (2006). Contribution of the type III secretion system (TTSS) to virulence of *Aeromonas salmonicida* subsp. salmonicida. Microbiology.

[52] Ebanks R.O., Knickle L.C., Goguen M., Boyd J.M., Pinto D.M., Reith M., Ross N.W. (2006). Expression of and secretion through the *Aeromonas salmonicida* type III secretion system. Microbiology.

[53] Marcoux P.É., Vincent A.T., Massicotte M.A., Paquet V.E., Doucet É.J., Hosseini N., Charette S.J. (2020). Systematic analysis of the stress-induced genomic instability of Type Three Secretion System in *Aeromonas salmonicida* subsp. salmonicida. Microorganisms.

[54] Burr S.E., Pugovkin D., Wahli T., Segner H., Frey J. (2005). Attenuated virulence of an *Aeromonas salmonicida* subsp. salmonicida type III secretion mutant in a rainbow trout model. Microbiology.

[55] Daher R.K., Filion G., Tan S.G.E., Dallaire-Dufresne S., Paquet V.E., Charette S.J. (2011). Alteration of virulence factors and rearrangement of pAsa5 plasmid caused by the growth of *Aeromonas salmonicida* in stressful conditions. Vet. Microbiol..

