# Supplementary material for: Genomic Analysis of Aeromonas salmonicida ssp. salmonicida Isolates Collected During Multiple Clinical Outbreaks Supports Association with a Single Epidemiological Unit
Source: Pathogens. 2024 Oct 17;13(10):908. doi: 10.3390/pathogens13100908 (PMC11510180; doi:10.3390/pathogens13100908)
Supplement: Supplementary file 1 [file pathogens-13-00908-s001.zip › pathogens-3262342-supplementary.pdf]

Supplementary File No 1

Tab.S1. Comparison of reference protein with samples (blastp).

Protein (% of Identity) / Sample_ID	ASA_2540	ASA_0509	ASA_1438	ASA_1267	OmpC	OmpA	OmpF
115-1	100	100	100	100	100	100	100
117BS-1	100	100	100	100	100	100	100
117ES-1	100	100	100	100	100	100	100
133-1	100	100	100	100	100	100	100
135-1	100	100	100	100	100	100	100
137-1	100	100	100	100	100	100	100
140-1-1	100	100	100	100	100	100	100
142-1	100	100	100	100	100	100	100
14f3-1	100	100	100	100	100	100	100
155-1	100	100	100	100	100	100	100
156-1	100	100	100	100	100	100	100
160-1	100	100	100	100	100	100	100
179-1	100	100	100	100	100	100	100
185-1	100	100	100	100	100	100	100
187-1	100	100	100	100	100	100	100
206-1	100	100	100	100	100	100	100
217-1-1	100	100	100	100	100	100	100
222-1	100	100	100	100	100	100	100
234-1	100	100	100	100	100	100	100

236-1	100	100	100	100	100	100	100
237-1	100	100	100	100	100	100	100
249-1	100	100	100	100	100	100	100
250-1	100	100	100	100	100	100	100
254-1-1	100	100	100	100	100	100	100
255-1	100	100	100	100	100	100	100
256-1	100	100	100	100	100	100	100
262-1	100	100	100	100	100	100	100
277-1	100	100	100	100	100	100	100
280-1	100	100	100	100	100	100	100
282-1	100	100	100	100	100	100	100
290-1	100	100	100	100	100	100	100
295-1	100	100	100	100	100	100	100
300-1	100	100	100	100	100	100	100
302-1	100	100	100	100	100	100	100
303-1	100	100	100	100	100	100	100
311-1	100	100	100	100	100	100	100
327-1	100	100	100	100	100	100	100
330-1	100	100	100	100	100	100	100
352-1	100	100	100	100	100	100	100
354-1	100	100	100	100	100	100	100
356-1	100	100	100	100	100	100	100
409-1	100	100	100	100	100	100	100
410-1	100	100	100	100	100	100	100
457-1	100	100	100	100	100	100	100
458-1	100	100	100	100	100	100	100
461-1	100	100	100	100	100	100	100
482A-1	100	100	100	100	100	100	100
54-1	100	100	100	100	100	100	100

57-1	100	100	100	100	100	100	100
71-1	100	100	100	100	100	100	100
78ES-1_1	100	100	100	100	100	100	100
8-1	100	100	100	100	100	100	100
F3-1	100	100	100	100	100	100	100
F4-1	100	100	100	100	100	100	100

A

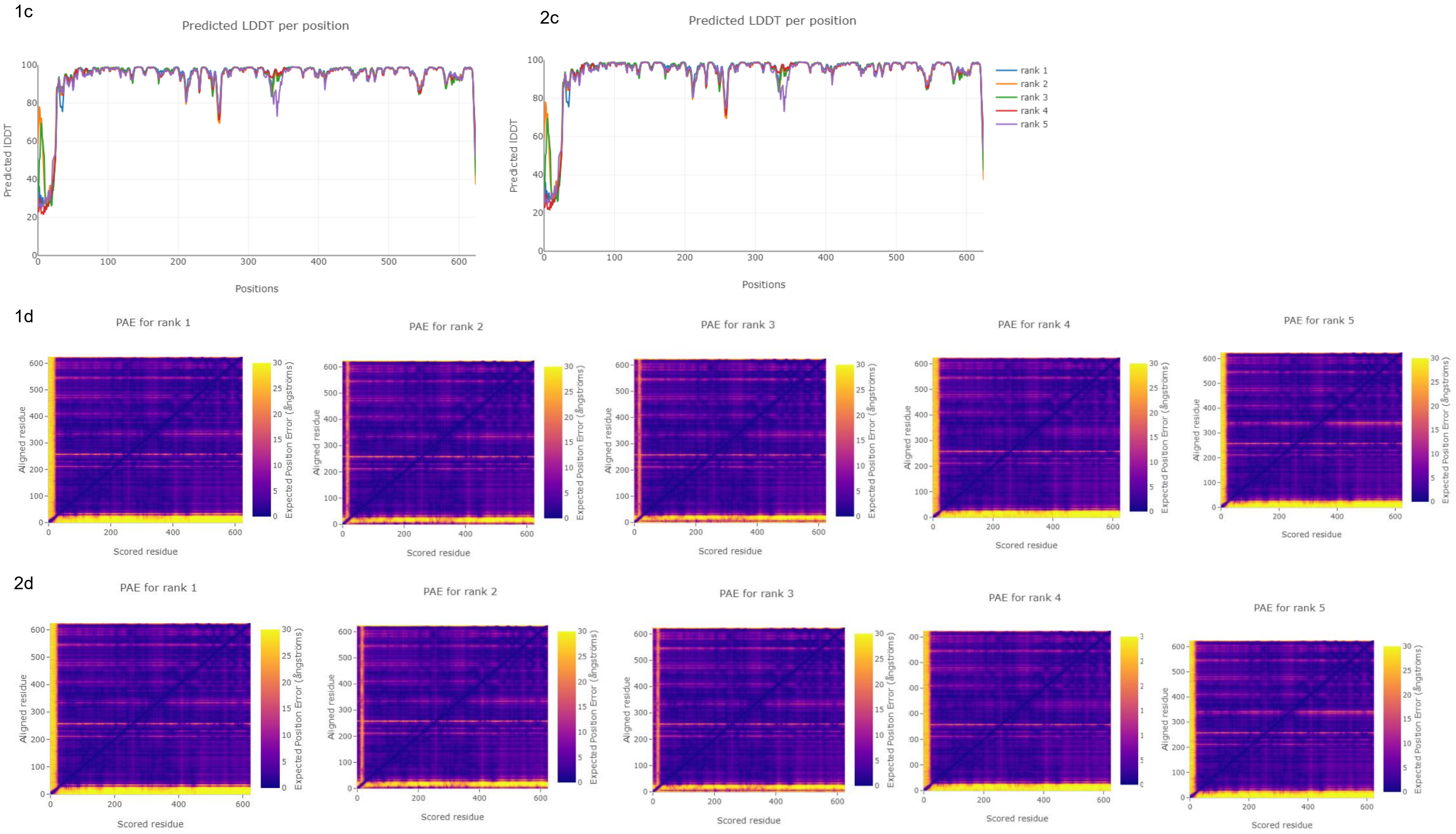
1c
Predicted LDDT per position
Predicted lDDT
Positions
2c
Predicted LDDT per position
rank 1
rank 2
rank 3
rank 4
rank 5
Predicted lDDT
Positions
1d
PAE for rank 1
PAE for rank 2
PAE for rank 3
PAE for rank 4
PAE for rank 5
Aligned residue
Scored residue
Expected Position Error (Ångströms)
2d
PAE for rank 1
PAE for rank 2
PAE for rank 3
PAE for rank 4
PAE for rank 5
Aligned residue
Scored residue
Expected Position Error (Ångströms)

B

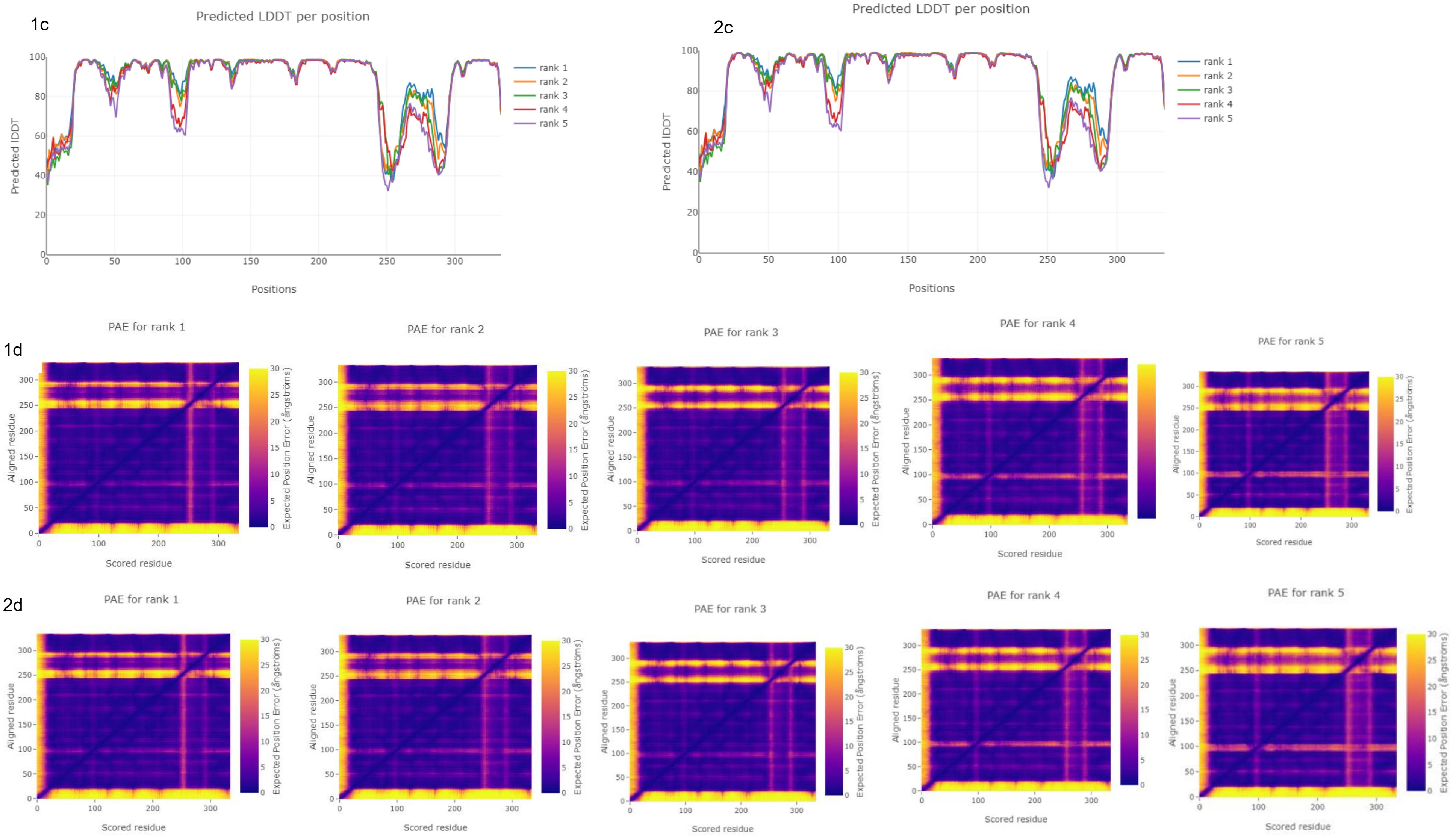

1c
Predicted LDDT per position
Predicted lDDT
Positions
rank 1
rank 2
rank 3
rank 4
rank 5
2c
Predicted LDDT per position
Predicted lDDT
Positions
rank 1
rank 2
rank 3
rank 4
rank 5
1d
PAE for rank 1
PAE for rank 2
PAE for rank 3
PAE for rank 4
PAE for rank 5
Aligned residue
Scored residue
Expected Position Error (Ångströms)
2d
PAE for rank 1
PAE for rank 2
PAE for rank 3
PAE for rank 4
PAE for rank 5
Aligned residue
Scored residue
Expected Position Error (Ångströms)

C

1c

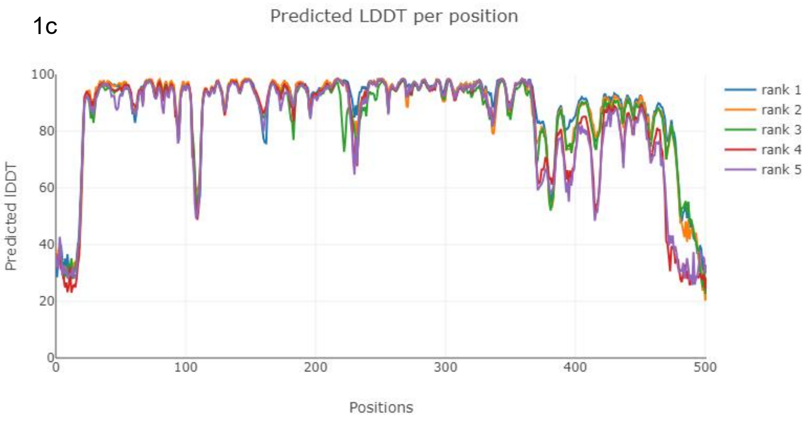
Predicted LDDT per position
Predicted lDDT
Positions
rank 1
rank 2
rank 3
rank 4
rank 5

2c

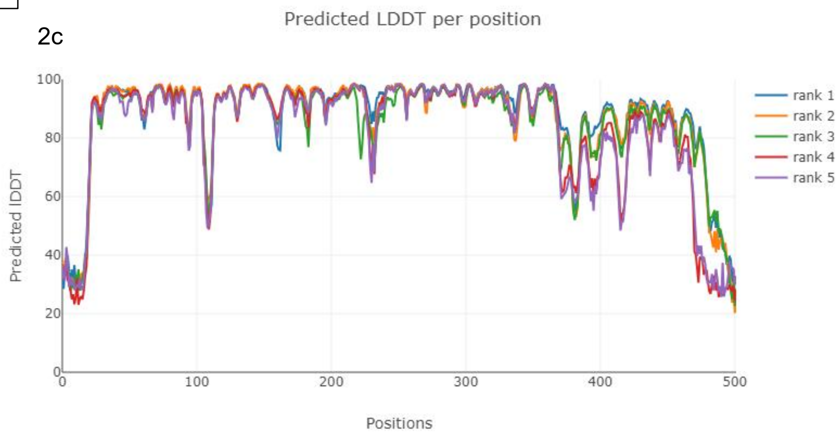
Predicted LDDT per position
Predicted lDDT
Positions
rank 1
rank 2
rank 3
rank 4
rank 5

1d

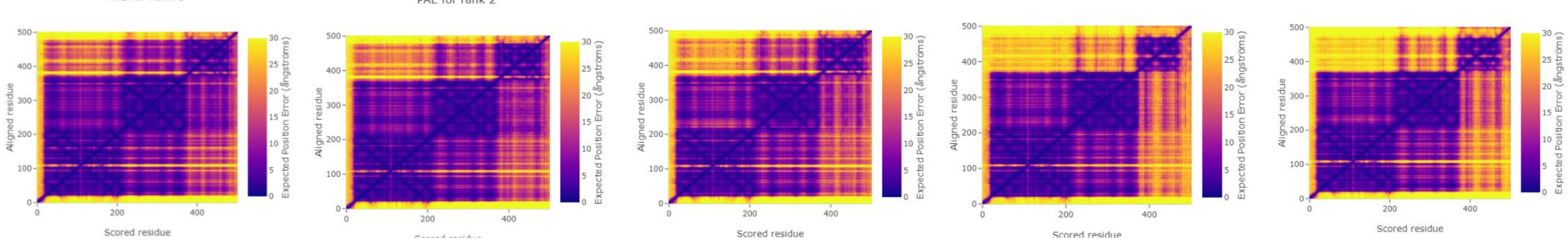
PAE for rank 1
PAE for rank 2
PAE for rank 3
PAE for rank 4
PAE for rank 5
Aligned residue
Scored residue
Expected Position Error (ångströms)

2d

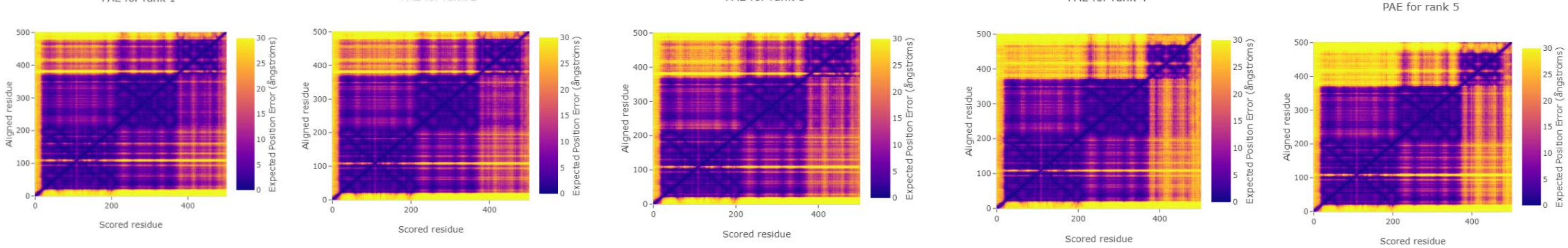
PAE for rank 1
PAE for rank 2
PAE for rank 3
PAE for rank 4
PAE for rank 5
Aligned residue
Scored residue
Expected Position Error (ångströms)

D

1c

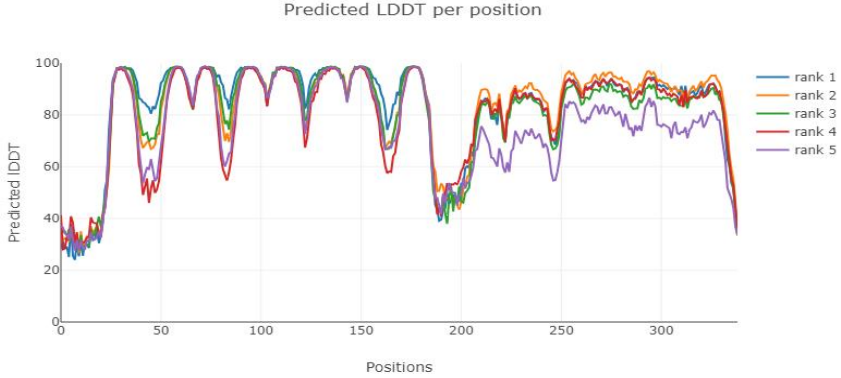
Predicted LDDT per position
Predicted lDDT
Positions
rank 1
rank 2
rank 3
rank 4
rank 5

2c

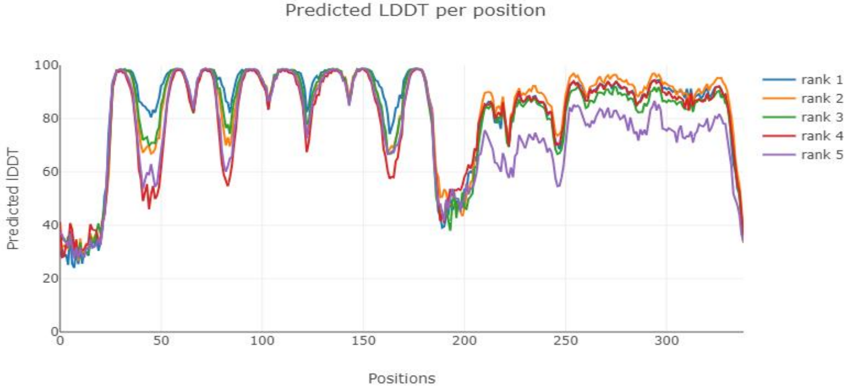
Predicted LDDT per position
Predicted lDDT
Positions
rank 1
rank 2
rank 3
rank 4
rank 5

1d

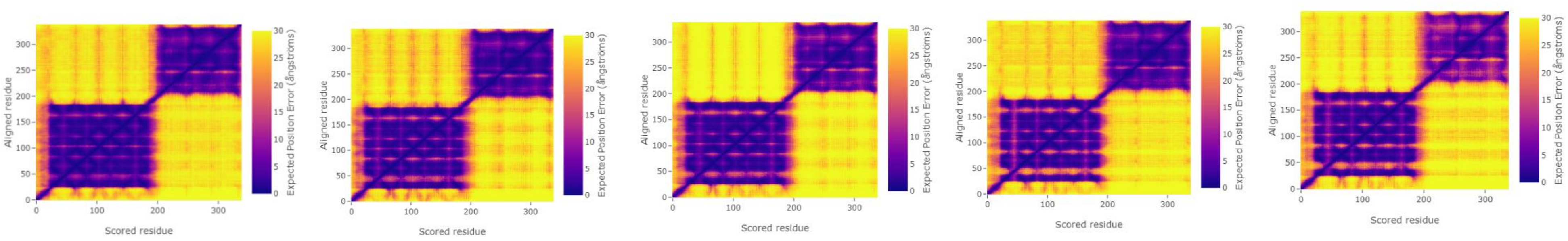
PAE for rank 1
PAE for rank 2
PAE for rank 3
PAE for rank 4
PAE for rank 5
Aligned residue
Scored residue
Expected Position Error (ångströms)

2d

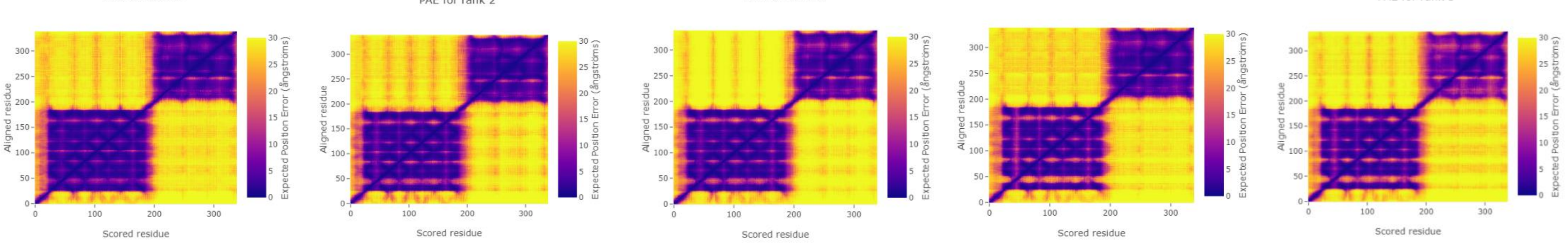
PAE for rank 1
PAE for rank 2
PAE for rank 3
PAE for rank 4
PAE for rank 5
Aligned residue
Scored residue
Expected Position Error (ångströms)

E

1c

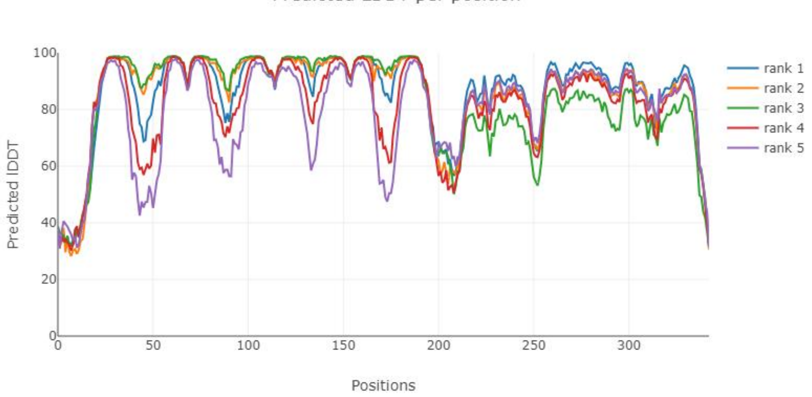
Predicted LDDT per position
Predicted lDDT
Positions
rank 1
rank 2
rank 3
rank 4
rank 5

2c

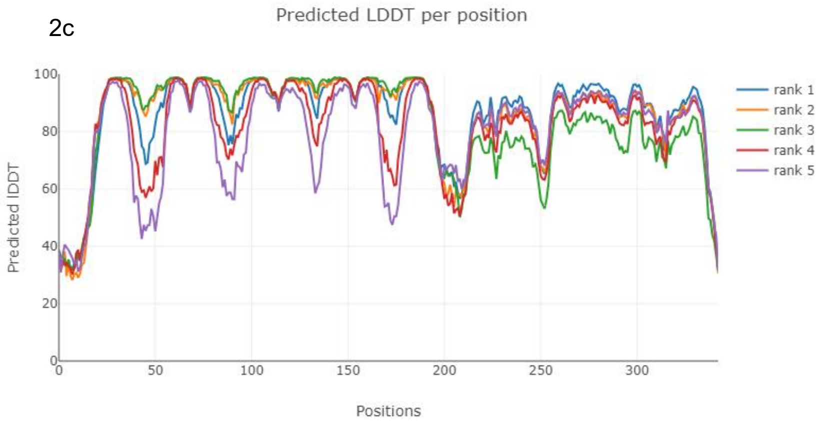
Predicted LDDT per position
Predicted lDDT
Positions
rank 1
rank 2
rank 3
rank 4
rank 5

1d

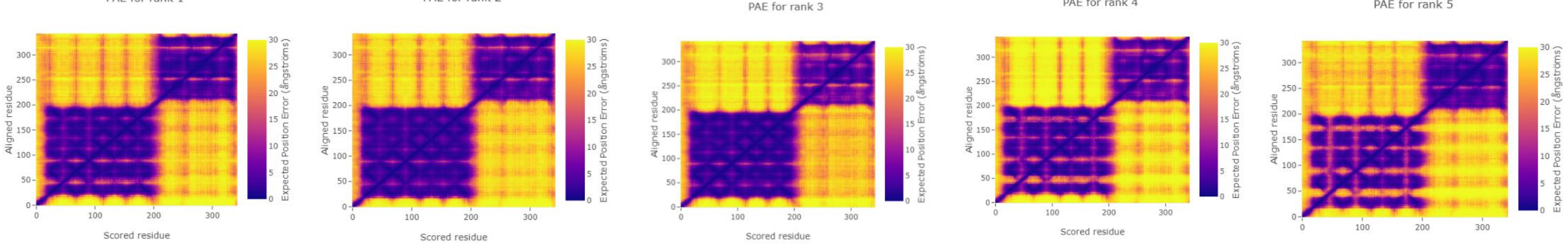
PAE for rank 1
PAE for rank 2
PAE for rank 3
PAE for rank 4
PAE for rank 5
Aligned residue
Scored residue
Expected Position Error (ångströms)

2d

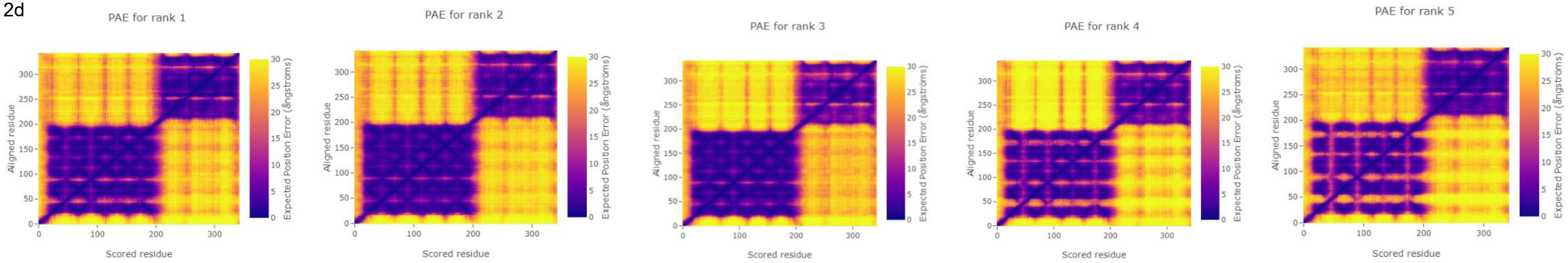
PAE for rank 1
PAE for rank 2
PAE for rank 3
PAE for rank 4
PAE for rank 5
Aligned residue
Scored residue
Expected Position Error (ångströms)

F

1c

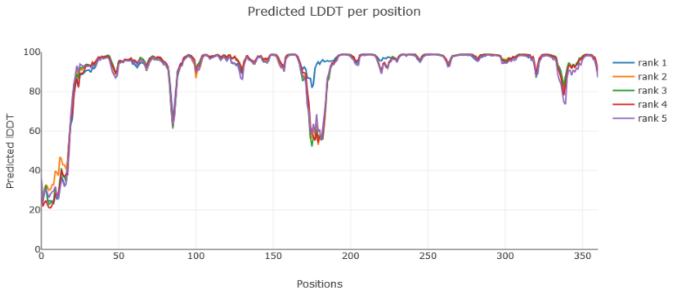
Predicted LDDT per position
Predicted lDDT
Positions
rank 1
rank 2
rank 3
rank 4
rank 5

2c

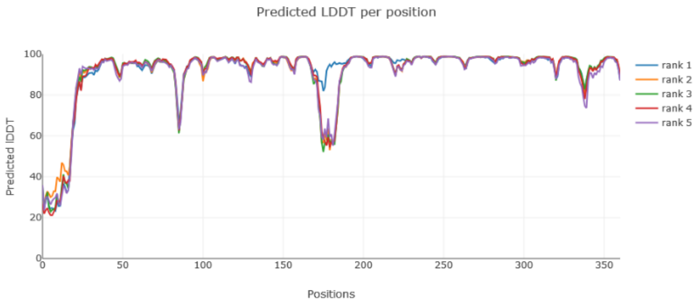
Predicted LDDT per position
Predicted lDDT
Positions
rank 1
rank 2
rank 3
rank 4
rank 5

1d

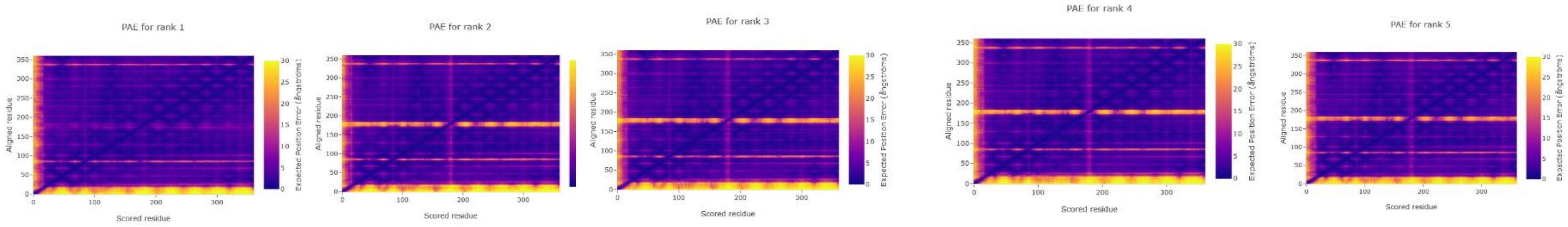
PAE for rank 1
PAE for rank 2
PAE for rank 3
PAE for rank 4
PAE for rank 5
Aligned residue
Scored residue
Expected Position Error (Ångströms)

2d

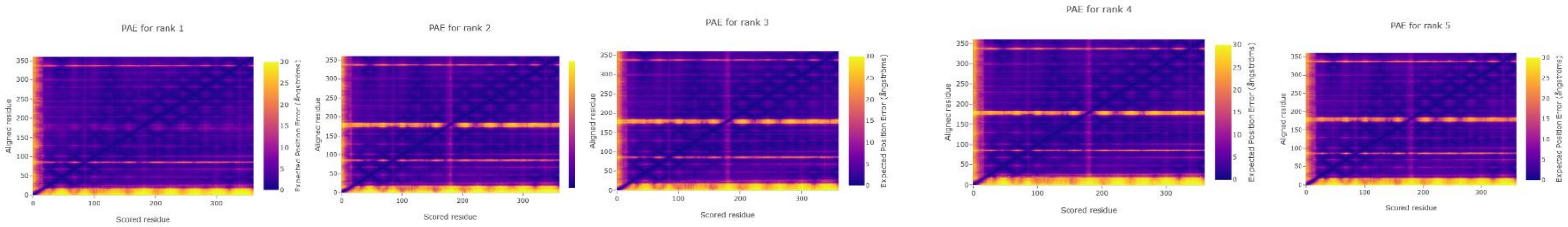
PAE for rank 1
PAE for rank 2
PAE for rank 3
PAE for rank 4
PAE for rank 5
Aligned residue
Scored residue
Expected Position Error (Ångströms)

G

1c

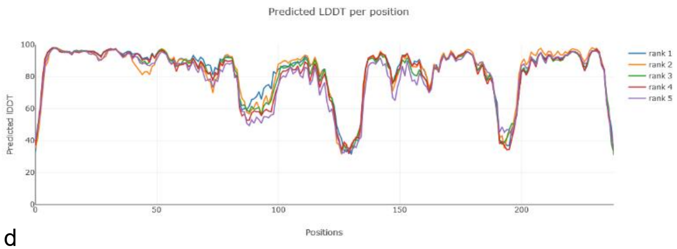
Predicted LDDT per position
Predicted lDDT
Positions
rank 1
rank 2
rank 3
rank 4
rank 5

2c

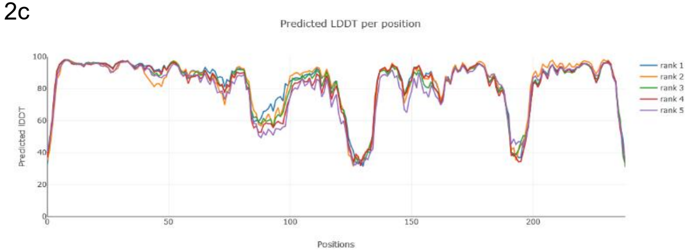
Predicted LDDT per position
Predicted lDDT
Positions
rank 1
rank 2
rank 3
rank 4
rank 5

1d

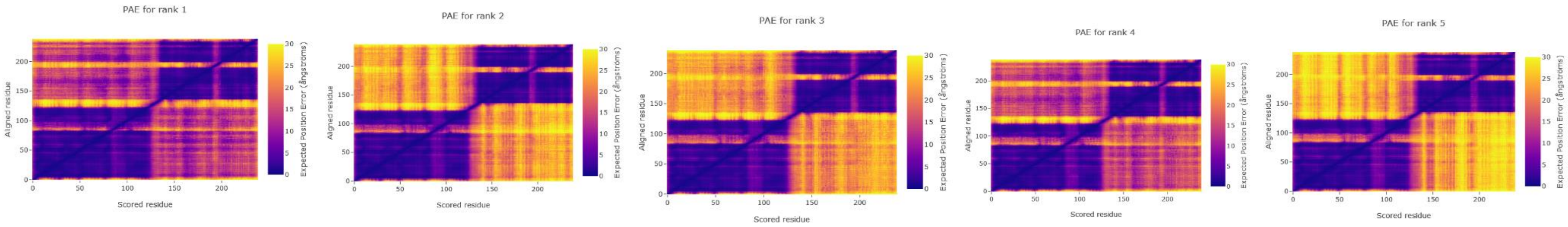
PAE for rank 1
PAE for rank 2
PAE for rank 3
PAE for rank 4
PAE for rank 5
Aligned residue
Scored residue
Expected Position Error (Ångströms)

2d

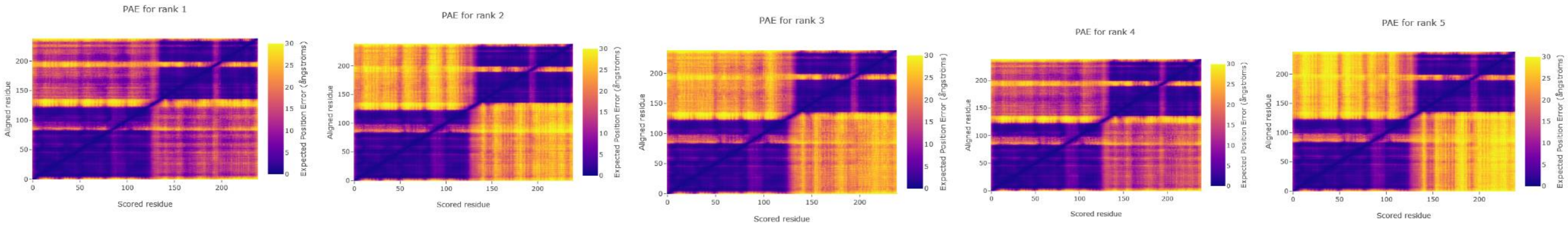
PAE for rank 1
PAE for rank 2
PAE for rank 3
PAE for rank 4
PAE for rank 5
Aligned residue
Scored residue
Expected Position Error (Ångströms)

H

1c

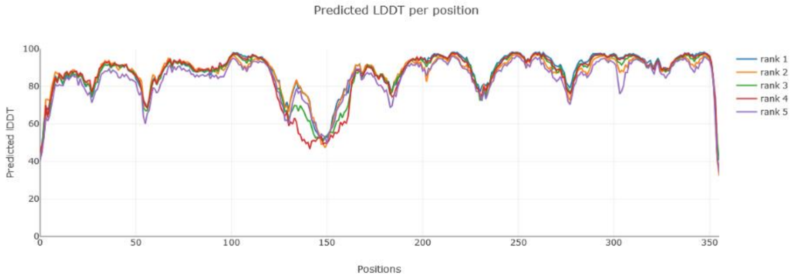

Predicted LDDT per position
Predicted lDDT
Positions
rank 1
rank 2
rank 3
rank 4
rank 5

2c

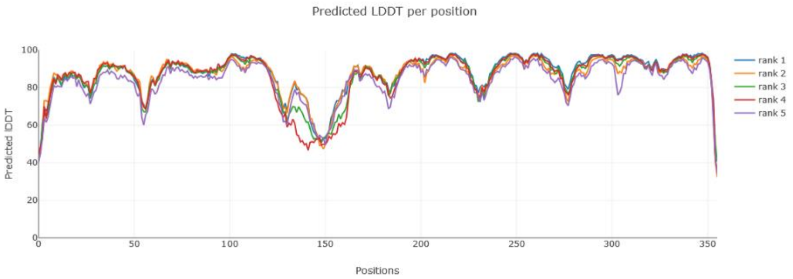

Predicted LDDT per position
Predicted lDDT
Positions
rank 1
rank 2
rank 3
rank 4
rank 5

1d

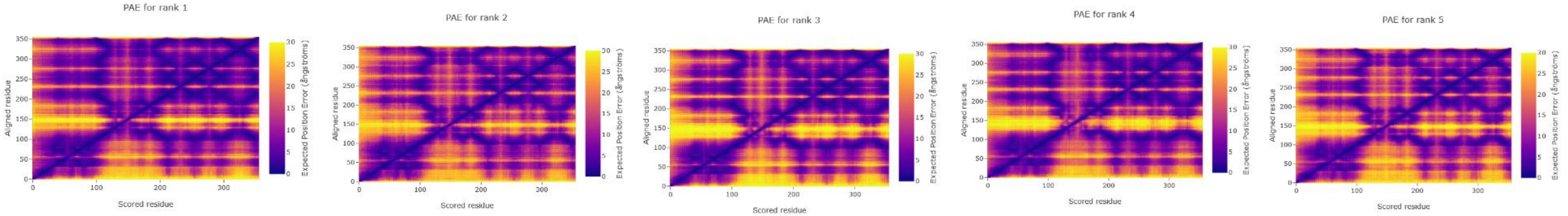

PAE for rank 1
PAE for rank 2
PAE for rank 3
PAE for rank 4
PAE for rank 5
Aligned residue
Scored residue
Expected Position Error (Ångströms)

2d

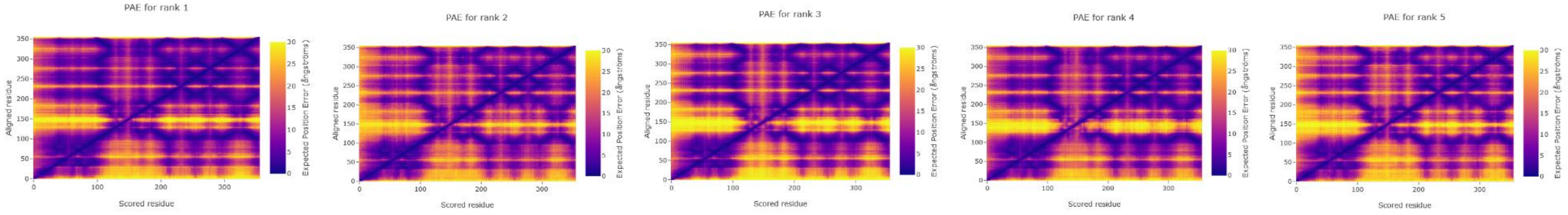

PAE for rank 1
PAE for rank 2
PAE for rank 3
PAE for rank 4
PAE for rank 5
Aligned residue
Scored residue
Expected Position Error (Ångströms)

I

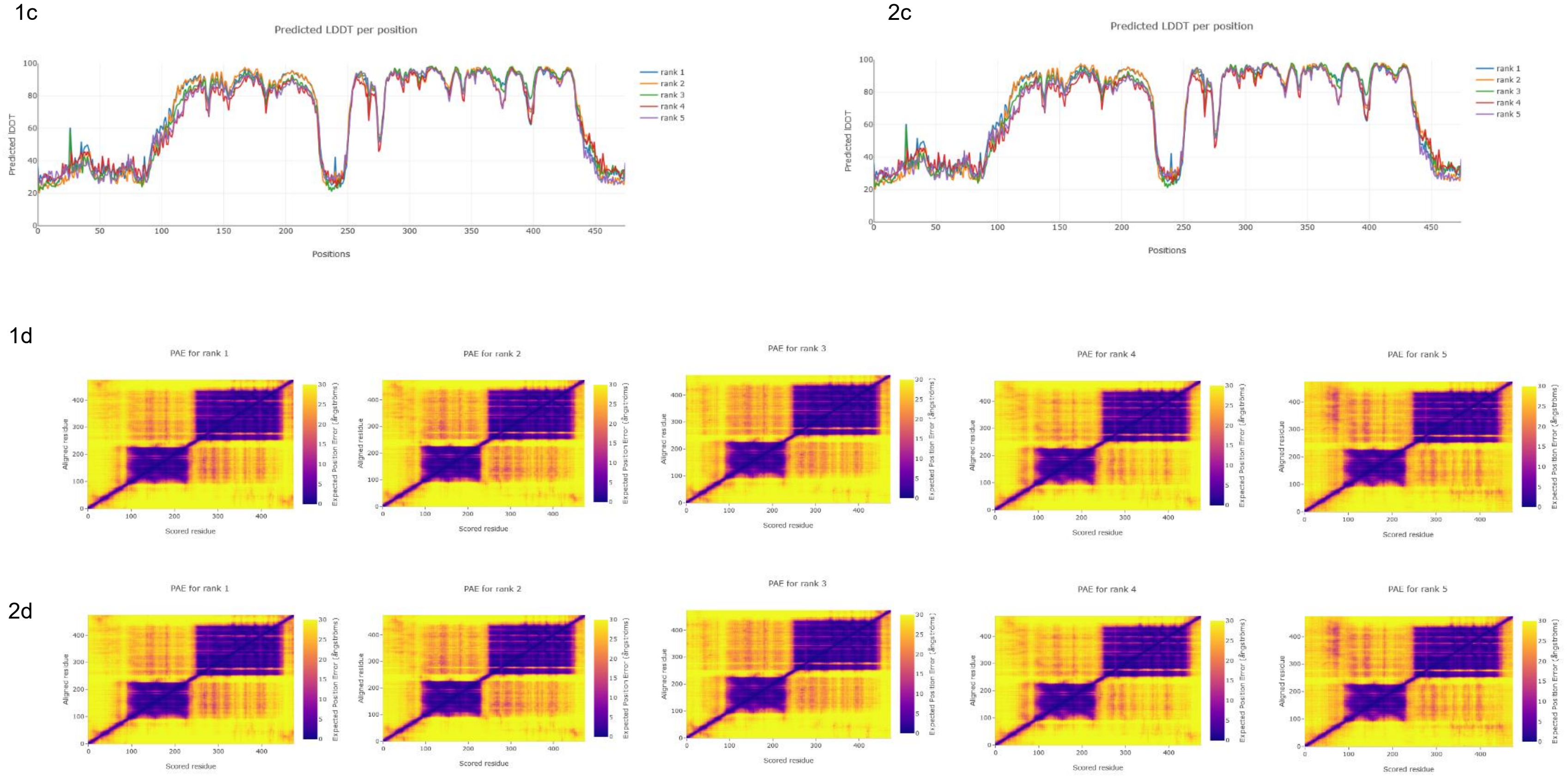

Fig. 1. Protein structure analysis between reference and selected sample (290-1) [where: A – ASA_2540; B – ASA_ 0509; C – ASA_1438; D – ASA_1267; E – OmpA; F – OmpC; G – OmpF; H – AscC; I – AexT; c – predicted LDDT per position; d – PAE; 1 – sample; 2 – reference]
